# Mixed evolutionary origins of endogenous biomass-depolymerizing enzymes in animals

**DOI:** 10.1186/s12864-018-4861-0

**Published:** 2018-06-20

**Authors:** Wai Hoong Chang, Alvina G. Lai

**Affiliations:** Nuffield Department of Medicine, University of Oxford, Oxford, OX3 7FZ UK

**Keywords:** Lignocellulose digestion, Comparative genomics, Glycoside hydrolase, Biomass, Biofuel, Metazoa, Evolution, Horizontal gene transfer

## Abstract

**Background:**

Animals are thought to achieve lignocellulose digestion via symbiotic associations with gut microbes; this view leads to significant focus on bacteria and fungi for lignocellulolytic systems. The presence of biomass conversion systems hardwired into animal genomes has not yet been unequivocally demonstrated.

**Results:**

We perform an exhaustive search for glycoside hydrolase (GH) genes from 21 genomes representing major bilaterian (Ecdysozoa, Spiralia, Echinodermata and Chordata) and basal metazoan (Porifera and Cnidaria) lineages. We also assessed the genome of a unicellular relative of Metazoa, *Capsaspora owczarzaki* and together with comparative analyses on 126 crustacean transcriptomes, we found that animals are living bioreactors at a microscale as they encode enzymatic suites for biomass decomposition. We identified a total of 16,723 GH homologs (2373 genes from animal genomes and 14,350 genes from crustacean transcriptomes) that are further classified into 60 GH families. Strikingly, through phylogenetic analyses, we observed that animal lignocellulosic enzymes have multiple origins, either inherited vertically over millions of years from a common ancestor or acquired more recently from non-animal organisms.

**Conclusion:**

We have conducted a systematic and comprehensive survey of GH genes across major animal lineages. The ability of biomass decay appears to be determined by animals’ dietary strategies. Detritivores have genes that accomplish broad enzymatic functions while the number of GH families is reduced in animals that have evolved specialized diets. Animal GH candidates identified in this study will not only facilitate future functional genomics research but also provide an analysis platform to identify enzyme candidates with industrial potential.

**Electronic supplementary material:**

The online version of this article (10.1186/s12864-018-4861-0) contains supplementary material, which is available to authorized users.

## Background

Increasing global demands for fossil fuels have led to investigations into alternative sources of renewable energy. As one of the most abundant reserves of photosynthetically fixed carbon on earth, plant lignocellulose provides a sustainable source of polysaccharides for fermentation to biofuels that may be harnessed to meet industrial and domestic needs. Acquiring simpler metabolites from lignocellulose is challenging due to the difficulties faced by enzymes in accessing the crystalline structure of cellulose that is encapsulated by lignin. Successful digestion of lignocellulosic tissues hence requires partial breakdown of lignin, which is achieved by fungi through the release of oxidizing free radicals that target woody cell wall components [1–3].

The traditional dogma that animals rely on endosymbionts for lignocellulose digestive capabilities because they lack endogenous cellulases has steered researchers to focus on fungi and bacteria. Industrial lignocellulosic bioprocessing mainly relies on enzymes isolated from the fungal species such as *Aspergillus* sp. and *Trichoderma reesei* for commercial preparations [4–7]. Moreover, much emphasis has been placed on the role of symbiotic intestinal microbes in the decomposition of plant biomass. For example, using metagenomics approaches, genes involved in lignocellulose digestion have been identified from gut commensal microbes in insects [8–10] and mammals [11, 12].

In recent years, multiple studies have begun to shed light on the presence of endogenous lignocellulolytic systems in animals, particularly in invertebrates [13–17]. One study proposed that cellulases from the glycoside hydrolase family 9 (GH9) could have been derived from an ancient metazoan ancestor [17] while another study discovered cellobiohydrolases from the GH7 family in a limnoriid crustacean species [16]. Yet, it is unknown whether cellulases are widespread across major metazoan lineages since systematic characterization of animal cellulases has not been performed despite it being crucial for the understanding of their diversity and evolution. Thus, it remains unanswered whether the ability to digest lignocellulose is dictated by the animals’ own genetic composition. Are these cellulase genes acquired from non-animal organisms or inherited vertically from our ancestors?

Taxonomic classification of all GH entries from the Carbohydrate-Active Enzymes (CAZy) database [18, 19] revealed that CAZy entries are skewed towards representatives from Bacteria with metazoan entries contributing to only 3.3% of all GH sequences. We address this major deficit of metazoan GH homologs by performing exhaustive screening for GHs from 21 metazoan genomes, which include basal animal lineages and a unicellular relative of Metazoa, followed by manual examination of sequences. Here we show that animal genomes encode cellulase genes encompassing broad lignocellulolytic functions. Given the observation of endogenous GH7 in crustaceans [16, 20], we further assessed 126 crustacean transcriptome datasets and identified over 300 GH7 genes from species represented across the broader Crustacea including those from basal classes: Branchiopoda and Copepoda. Phylogenetic analyses of animal cellulases revealed that these genes either have ancient origins or were acquired horizontally from bacteria or fungi.

## Results and discussion

### Taxonomic classification of glycoside hydrolases from CAZy and phylogenetic analysis of metazoan CAZy homologs

**Fig. 1:**
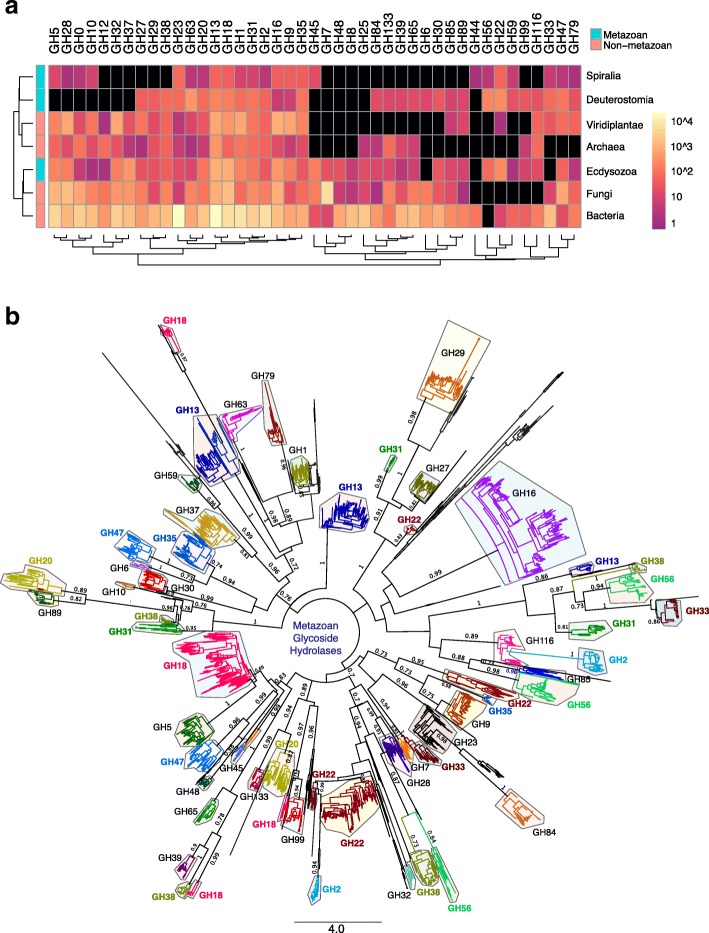
Taxonomic distribution and phylogenetic analysis of CAZy glycoside hydrolases (GHs) in metazoans. **a** Heatmap depicts 43 GH families containing metazoan representatives. The number of GH genes within each family and taxon are color-coded according to a log10 scale. Dendrograms present clustering of taxa (rows) and GH families (columns) based on hierarchical clustering with Euclidean distance metric and average linkage. Black boxes denote absent members within a particular GH family. **b** Consensus phylogeny of animal GHs generated from Bayesian and maximum likelihood analyses with GH families color coded. Posterior probability support of > 0.7 and bootstrap support of > 70% are denoted as node labels. Scale bar correspond to substitution per site. The names of polyphyletic GH families are indicated in bold face and colour coded

We retrieved 188,668 lignocellulolytic glycoside hydrolase (GH) sequences from the CAZy database [18] and assessed their taxonomic distribution since CAZy does not currently provide taxonomic classification for its sequences beyond the highest rank of Archaea, Bacteria and Eukaryota domains. CAZy GH entries are dominated by Bacteria sequences (81%) with the rest distributed across other major taxa: Archaea (1.1%), Viridiplantae (3.8%), Fungi (10.1%) and Metazoa (3.3%; 2.1% from Ecdysozoa, 0.2% from Spiralia and 1% from Deuterostomia) (Additional file 1: Figure S1; Additional file 2: Figure S2). Proteobacteria makes up 48% of bacterial entries with a majority of genes arising from Gram negative Enterobacterales (Additional file 2: Figure S2; Additional file 3: Figure S3). Most of the remaining bacteria GHs are found within the Bacilli class of (Terrabacteria Additional file 2: Figure S2; Additional file 3: Figure S3). Archaeal GHs are classified into two main superphyla: Euryarchaeota and TACK (uniting Thaumarchaeota, Aigarchaeota, Crenarchaeaota and Korarchaeota phyla) [21] where the former includes methanogens (Methanomicrobia) found in intestines (Additional file 2: Figure S2; Additional file 4: Figure S4). Fungal GHs overshadow eukaryotic entries with 49 and 50% of fungal GHs observed in species from the Saccharomycotina and Pezizomycotina subphyla of Ascomycota (Additional file 2: Figure S2; Additional file 5: Figure S5). Flowering eudicot land plants (Embryophyta) contribute to 98% of GHs in Viridiplantae (Additional file 2: Figure S2; Additional file 6: Figure S6). Of the 127 GH families, we observed that only 43 families have metazoan representatives while 84 families do not (Fig. 1a, Additional file 1: Figure S1). We identified 6299 metazoan GH genes where by far the most abundant sequences originating from Ecdysozoa, with a particular concentration in insects (Additional file 2: Figure S2A; Additional file 7: Figure S7). As evident from the metazoan GH tree generated using maximum likelihood and Bayesian methods [22, 23], GHs are resolved into structurally unrelated families with a majority of GH families exhibiting monophyly (Fig. 1b), suggesting that the ability to degrade cellulose may have evolved independently on multiple occasions. Notably, GH2, GH13, GH18, GH20, GH22, GH31, GH33, GH35, GH38, GH47 and GH56 are polyphyletic (Fig. 1b), which could be explained by the existence of multi-domain architectures found in a number of cellulolytic enzymes to allow multiple catalytic activities to synergistically interact [24, 25].

### Classification of a complete set of glycoside hydrolases from 21 metazoan genomes representing major animal taxa

**Fig. 2:**
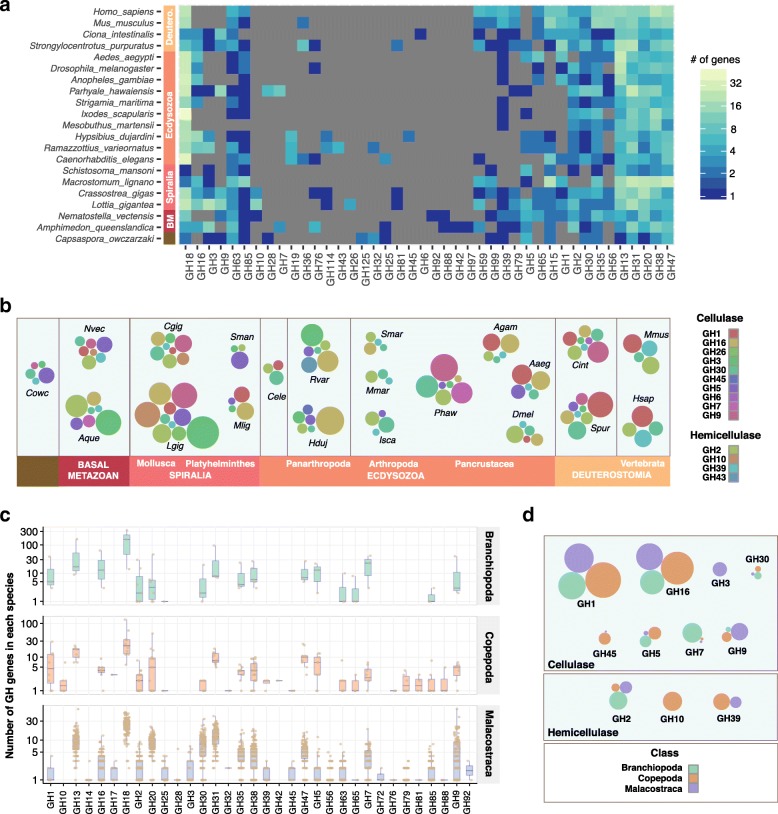
GH families, including those with cellulase function, identified from 21 metazoan genomes and 126 crustacean transcriptomes. **a** Heatmap depicts 42 GH families encoded by animal genomes presented as complete linkage clustering of GH families (columns). The number of GH genes within each family are color-coded according to a log2 scale. Grey boxes denote absent members within a particular GH family. **b** Cellulase GHs from each animal are depicted as a bubble chart. Bubble sizes are proportional to gene abundance and GH families are color-coded. Species names are abbreviated by taking the first letter of genus name and first three letters of species name; *Homo sapiens* (*Hsap*)*.* Full species names are available in Additional file 8: Table S1. **c** GH families identified from crustacean transcriptomes are illustrated as box-plots with jittered points representing the number of GH genes from each crustacean species. **d** Bubbles represent the proportion of cellulases identified across three crustacean classes. The bubble size is normalized to number of species analyzed to account for the differential sample size

Enzymatic decomposition of lignocellulose is achieved by GHs with cellulase and hemicellulase activities observed in twenty families (GH1, GH2, GH3, GH5, GH6, GH7, GH8, GH9, GH10, GH12, GH16, GH26, GH30, GH39, GH43, GH44, GH45, GH48, GH51 and GH74) [19, 26]. More often than not, it has been assumed that cellulase activity found in animals is attributed to microbial agents located in intestinal tracts such as those observed in termites and ruminants [14, 27, 28]. Reports on cellulase-containing GHs in animals have been limited to a few observations on GH7 and GH9 families [13, 16, 29, 30]. Fungi possess all cellulase families except for GH44 (Fig. 1a; Additional file 5: Figure S5). As Fungi are the closest relative to metazoans, we hypothesize that the last common ancestor of Opisthokonta would have possessed most if not all of the nineteen cellulase-encoding GHs. Our whole genome analyses of GHs in 21 animals representing major lineages revealed a comprehensive array of lignocellulose-degrading enzymes categorized into 60 GH families (Additional file 8: Table S1; Additional file 9: Table S2). For visualization purposes, the heatmap in Fig. 2a depicts 42 of the 60 families as sub-families are collapsed and families with less than 3 members are not included. As mentioned previously, 20 GH families possess cellulase function and 14 families are found in animals’ genomes (Fig. 2b), refuting the common opinion that endogenous cellulases are a rarity in animals. Of the 2373 animal GH genes identified, the most abundantly represented GH families are chitinases (GH18; 17%), α-glucosidases (GH31; 10%), α-amylases (GH13; 9%) and α-mannosidases (GH38; 8%) (Additional file 2: Figure S2C). As the closest unicellular relative of metazoans, the filasterean *Capsaspora owczarzaki* contains 45 GH genes categorised into 21 families, five of which are cellulases (Fig. 2a, b), suggesting that there is a requirement for biomass-degrading enzymes in the life history of our single-celled ancestor. This view is further reinforced with the discovery of 106 and 81 GH genes in early branching metazoans, the sponge (*Amphimedon queenslandica*) and the starlet sea anemone (*Nematostella vectensis*) respectively (Fig. 2a). These primitive marine animals have retained seven cellulase families (Fig. 2b).

Within Bilateria, molluscs have among the most diverse sets of cellulases; ten families from the gastropod owl limpet (*Lottia gigantea*) and eight from the Pacific oyster (*Crassostrea gigas*), while a significant reduction is observed in other members of Spiralia, i.e. Platyhelminthes (Fig. 2a, b). Further cellulase loss is seen in the ecdysozoan lineage leading to Nematoda with only three cellulase families found in *Caenorhabditis elegans* (Fig. 2a, b). At the base of Panarthropoda, tardigrades have 6 cellulase families but this level of diversity is not observed in arachnids and centipede (Fig. 2a, b). Within Pancrustacea, the amphipod crustacean *Parhyale hawaiensis* has 156 GH genes, seven of which possess cellulase function signifying an adaptation to a detritivorous diet (Fig. 2a, b) [31]. Dipterans (fruit fly and mosquitoes), however, have reduced number of GHs and cellulases (Fig. 2a, b). The closest known relative of deuterostomian chordates, the sea urchin, has 142 GH genes encompassing 8 cellulase families (Fig. 2a, b). This level of cellulase diversity is shared in another marine invertebrate deuterostome, the ascidian *Ciona intestinalis* (Fig. 2a, b). Lignocellulose decomposition mechanisms that shaped the genetic contents of sea urchin and ascidian are not extended to vertebrates (human and mouse), where the latter have markedly reduced cellulase-containing GHs (Fig. 2a, b). It is striking that cellobiohydrolases (GH5, 6 and 9), although found in sea urchin and/or ascidian, were absent from vertebrates.

### Innovation of endogenous GH7 and GH9 homologs in Crustacea

Given a previous report on endogenous cellulases in the isopod crustacean *Limnoria quadripunctata* [16], we investigated the diversity of GH families in 126 crustacean species found across the broader Crustacea including basal classes (Branchiopoda and Copepoda) and economically important food crop species from Malacostraca (Additional file 8: Table S1). We identified 14,350 GH orthologs from crustacean transcriptome data sets that are classified into 34 GH families (eleven of which are cellulases) according to CAZy nomenclature (Fig. 2c, d; Additional file 10: Table S3). Thirty GH families are retained in the Multicrustacea lineage uniting Malacostraca and Copepoda (Fig. 2c). Only 18 GH families were identified in branchiopod transcriptomes (Fig. 2c). Although it still remains to be proven how reliable the number of GH families will be to provide support for the Allotriocarida clade with branchiopods and insects as members [32, 33], it is intriguing to note that 18 families are similarly observed in both taxa (Fig. 2a, c). GH7 enzymes have cellobiohydrolase activities important for effective breakdown of cellulose [34]. More importantly, we identified 318 GH7 genes in crustaceans along with genes from two other groups of cellobiohydrolases (770 GH9 genes and 158 GH5 genes) (Fig. 2c; Additional file 11: Figure S8), suggesting that crustaceans may have had superior autonomy for biomass decomposition than previously appreciated.

### Animal cellulases either have an ancient origin or were acquired recently through horizontal gene transfer

**Fig. 3:**
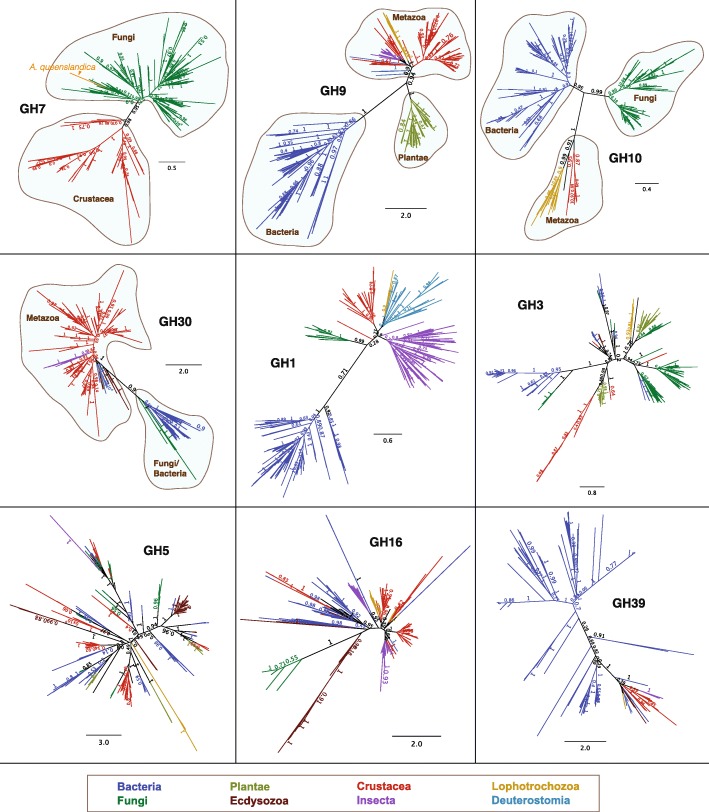
Vertical and horizontal transmission of animal cellulases. Unrooted tree topologies are supported by Bayesian and maximum likelihood analyses. Branches are color-coded according to taxonomic affiliation. GH7, GH9, GH10 and GH30 phylogenies support monophyletic metazoan groups while GH1, GH3, GH5, GH16, GH39 do not

The hypothesis that cellulase GHs in animals have emerged as functional life history adaptations is valid, despite their equivocal evolutionary origins. Two mechanisms can best explain the presence of animal cellulases: (1) intermittent acquisition of genes horizontally from bacteria or fungi; and (2) vertical inheritance of genes from a metazoan ancestor followed by differential gene loss in many extant taxa. Our examination of metazoan cellulase homologs revealed that GH1, GH3, GH5, GH16 and GH39 are likely derived from bacteria and/or fungi, perhaps through associations with intestinal microbiota (Fig. 3). This is consistent with the notion that most animals lack cellulases derived from an ancient ancestor but rather acquire genes through horizontal gene transfer (HGT) [35, 36]. HGT occurs more frequently for operational genes such as those involved in housekeeping or in driving simple enzymatic processes [37]. It is therefore possible for GHs involved in carbohydrate decomposition to be horizontally transferred since they are not typically members of large genetic networks. However, HGT alone cannot account for the evolution of all metazoan cellulases. Phylogenetic analyses of GH7, GH9, GH10 and GH30 revealed branching patterns recapitulating species tree with support for monophyly in metazoan homologs (Fig. 3). With the exception of GH7 from *A. queenslandica*, our analyses suggest that these genes are likely to be inherited vertically over several hundred million years from a metazoan ancestor rather than originating from recent HGT events. Interestingly, the aforementioned GH families include enzymes with diverse substrate preferences (cellobiohydrolases, endoglucanases, glucosidases and xylosidases), which point to the prospect of metazoan homologs related by vertical descent to collectively accomplish a wide range of biomass decomposition functions.

### Nutritional strategy underpins the diversity of animal cellulases

**Fig. 4:**
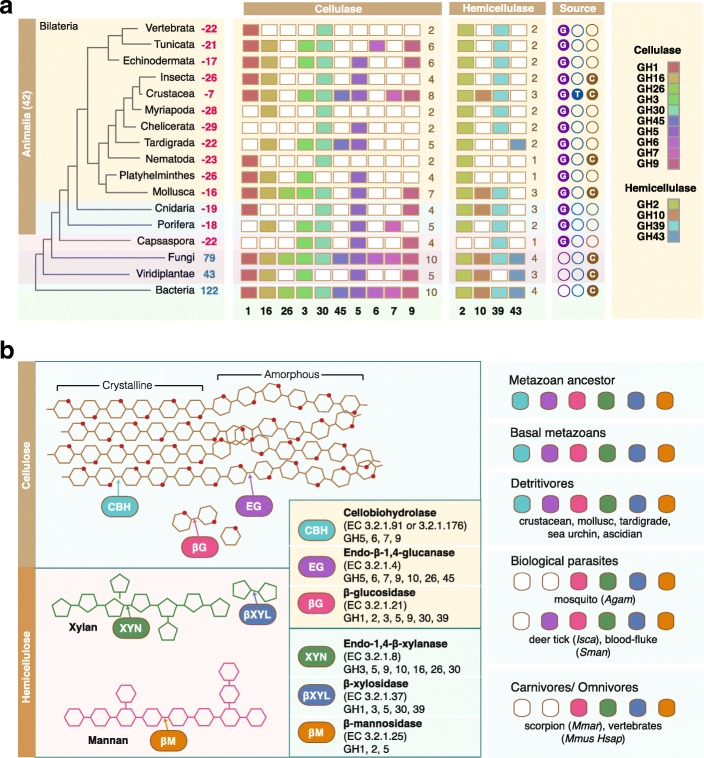
Evolution of lignocellulose decomposition machineries in animals. **a** Comparison of cellulases across the Tree of Life with focus on animals. The number of GH families lost from each taxon are denoted in red beside taxon labels. The number of GH families present in non-animal taxa are denoted in blue. Color boxes indicate the presence of certain cellulase family members while empty boxes indicate the loss of particular members. Data sources used for interpretation are depicted as colored circles; G = genome, T = transcriptome, C = CAZy. **b** The presence of lignocellulolytic enzymes is related to animal life history and dietary habits. Endoglucanases hydrolyse internal cellulose bonds while cellobiohydrolases cleave cellobiose units at chain ends. Glucosidases hydrolyse cellobiose units to release glucose. Xylan and mannan hemicellulose require xylosidases and mannosidases for their degradation. Animals that adopt detritivorous diets rich in plant biomass possess complete enzymatic suites for efficient lignocellulose decomposition and this is not seen in animals that adopt more specialized diets. Species names are abbreviated with full species names available in Additional file 8: Table S1

The prevalence of cellulases in animals suggests that these enzymes play fundamental roles in depolymerising lignocellulose found in their food sources. Three classes of GHs are required for the complete decomposition of lignocellulose [38]. Endoglucanases break down cellulose chains to make them accessible, cellobiohydrolases degrade cellulose chains to generate cellobiose and finally β-glucosidases catalyze the hydrolysis of glycosidic bonds to release glucose. Hemicellulases are also required to degrade polysaccharides namely mannans and xylans. Non-animal decomposers such as bacteria and fungi that thrive on decaying material have the most comprehensive array of cellulases (Fig. 4a, Additional file 1: Figure S1). Since the metazoan ancestor possesses at least 14 cellulase families, one could speculate that primitive animals may have maintained biomass-rich diets (Fig. 4). Aquatic ecosystems contain vast quantities of plant biomass that provides sustenance for a range of aquatic organisms. It is perhaps not surprising that benthic invertebrates or detritus feeders such as molluscs, crustaceans, sea urchin and ascidian would evolve enzymatic suites compatible with a diet rich in lignocellulose (Fig. 4a). Indeed, this group of animals harbor cellulases with broad enzymatic range, which supports the notion that a combination of endoglucanases and cellobiohydrolases would increase the efficiency of lignocellulose digestion (Fig. 4b) [34, 39]. Animals that have evolved specialized diets (omnivory or carnivory) or those that have adopted a parasitic lifestyle lack cellobiohydrolases or endoglucanases altogether (Fig. 4).

## Conclusion

In summary, we identified the putative full set of 2373 GH genes encoded by 21 genomes representing major bilaterian evolutionary lineages, basal metazoans and a unicellular relative of animals. Remarkably, animal genomes encode 14 GH families with cellulase functions and the diversity of cellulases appears to be related to animals’ dietary strategy that may facilitate greater autonomy for lignocellulose decomposition. Our analyses on crustacean transcriptomes revealed that endoglucanases and cellobiohydrolases are widespread in these species, which may in part, explain why these animals could survive on a detritivorous diet rich in plant biomass. GH7 from the isopod crustacean *L. quadripunctata* is shown to exhibit a number of striking features that are superior to fungal enzymes such as tolerance to high salt and enzyme denaturing conditions [20]. Although it remains to be determined whether the 318 crustacean GH7 homologs identified in study possess unique enzymatic capabilities, it is possible that these features are retained through adaptations to the marine environment. The natural cellulase diversity in animals may make inroads into current research focusing on optimizing enzymatic cocktails and hydrolysis strategies for industrial biomass conversion processes.

## Methods

### Taxonomic assignment of CAZy dataset

The 188,668 glycoside hydrolase (GH) sequences were retrieved from the CAZy database (http://www.cazy.org/) where information on species names are available [18, 26, 40–42]. Taxonomic assignment of each GH sequences based on species names was performed using a taxonomy toolkit, TaxonKit (http://bioinf.shenwei.me/taxonkit/). Sankey diagrams were generated using RAWGraphs (https://rawgraphs.io/) [43] to enable the visualization of taxonomic hierarchies within specific lineages.

### Identification of GH genes from metazoan genomes and crustacean transcriptomes

Reference proteomes of fully sequenced genomes were obtained from Uniprot (http://www.uniprot.org/proteomes/) and accession numbers are provided in Additional file 8: Table S1. Crustacean transcriptome datasets available at the time of manuscript preparation were retrieved from the European nucleotide archive (https://www.ebi.ac.uk/ena) with accession numbers provided in Additional file 8: Table S1. All 188,668 CAZy sequences were used as queries to identify GH homologs in animals. We used multiple basic local alignment search tool (BLAST)-based approaches such as BLASTp and tBLASTn with blocks substitution matrices BLOSUM45 and BLOSUM62 to allow sufficient sensitivity to identify distant GH homologs. BLAST results were subsequently combined and unique hits were filtered by e-value of < 10^− 5^, best reciprocal BLAST hits against the GenBank non-redundant (nr) database and redundant transcripts having at least 98% identity were collapsed using CD-HIT (https://github.com/weizhongli/cdhit). We then utilized HMMER hmmscan employing hidden Markov models (HMM) profiles [44] to scan for the presence of Pfam domains commonly present in GH proteins [45] on the best reciprocal nr BLAST hits to compile a final non-redundant set of animal GH homologs. Pfam annotations and associated e-values are provided in Additional file 9: Table S2 and Additional file 10: Table S3. Fasta file of GH sequences are provided in Additional files 12 and 13. Heatmaps were generated using the pheatmap package [46] in R and bubble charts were generated using RAWGraphs [43].

### Multiple sequence alignment and phylogenetic tree construction

Nucleotide sequences obtained from transcriptome analyses were translated to the correct frame using TransDecoder (version 5) [47]. Multiple sequence alignments of GH protein sequences obtained from CAZy, genome and transcriptome analyses were performed using MAFFT [48]. Phylogenetic trees were constructed from MAFTT alignments using RAxML [22] for maximum likelihood trees and MrBayes [49] for Bayesian trees. For maximum likelihood trees, the Whelan and Goldman (WAG) model of amino acid evolution [50] was used with 1000 bootstrap replications; four substitution rate categories were allowed with gamma distribution parameters estimated from the dataset. Bayesian analyses were performed on the multiple sequence alignments using a mixed amino acid substitution model; one tree was sampled for every 100 generations with the first 1000 trees discarded as burn-in. Both methods yielded trees with the same topology. Geneious was used to generate graphical representations of Newick trees [51].

## Additional files

Additional file 1: **Figure S1.** Heatmap depicts 84 GH families identified from CAZy that do not have metazoan representatives. The number of genes within each GH family and taxon are color-coded according to a log10 scale. Dendrograms present clustering of taxa (columns) and GH families (rows) based on hierarchical clustering with Euclidean distance metric and average linkage. Black boxes denote absent members within a particular GH family. (PDF 143 kb)

Additional file 2: **Figure S2.** Distribution of GH families retrieved from the CAZy database and identified in this study from metazoan genomes. **(A)** Pie charts represent the proportion of CAZy GH genes grouped according to taxa and GH families. **(B)** Proportion of CAZy GH genes within selected taxa are depicted. **(C)** The proportion of GH families identified from metazoan genomes are represented as pie charts grouped by species and by GH family. Numbers alongside pie charts in parentheses represent the total number of sequences. (PDF 1060 kb)

Additional file 3: **Figure S3.** Taxonomic Sankey diagram of CAZy glycoside hydrolases from Bacteria. (PDF 2021 kb)

Additional file 4: **Figure S4.** Taxonomic Sankey diagram of CAZy glycoside hydrolases from Archaea. (PDF 869 kb)

Additional file 5: **Figure S5.** Taxonomic Sankey diagram of CAZy glycoside hydrolases from Fungi. (PDF 1716 kb)

Additional file 6: **Figure S6.** Taxonomic Sankey diagram of CAZy glycoside hydrolases from Viridiplantae. (PDF 997 kb)

Additional file 7: **Figure S7.** Taxonomic Sankey diagram of CAZy glycoside hydrolases from Metazoa. (PDF 1211 kb)

Additional file 8: **Table S1.** List of accession numbers for species used in this study. (XLSX 23 kb)

Additional file 9: **Table S2.** Summary of glycoside hydrolase annotations in metazoan genomes. (XLSX 86 kb)

Additional file 10: **Table S3.** Summary of glycoside hydrolase annotations in crustacean transcriptomes. (XLSX 468 kb)

Additional file 11: **Figure S8.** Heatmap illustrating the abundance of GH genes identified from 126 crustacean species. The number of GH genes within each family and taxon are color-coded according to a log2 scale. Dendrograms present clustering of species (rows) and GH families (columns) based on hierarchical clustering with Euclidean distance metric and average linkage. Black boxes denote absent members within a particular GH family. (PDF 441 kb)

Additional file 12: Fasta file of glycoside hydrolase sequences identified from metazoan genomes. (TXT 1687 kb)

Additional file 13: Fasta file of glycoside hydrolase sequences identified from crustacean transcriptomes. (TXT 25708 kb)
