# Supplementary figures and images for: Mixed evolutionary origins of endogenous biomass-depolymerizing enzymes in animals
Source: BMC Genomics. 2018 Jun 20;19:483. doi: 10.1186/s12864-018-4861-0 (PMC6011409; doi:10.1186/s12864-018-4861-0)

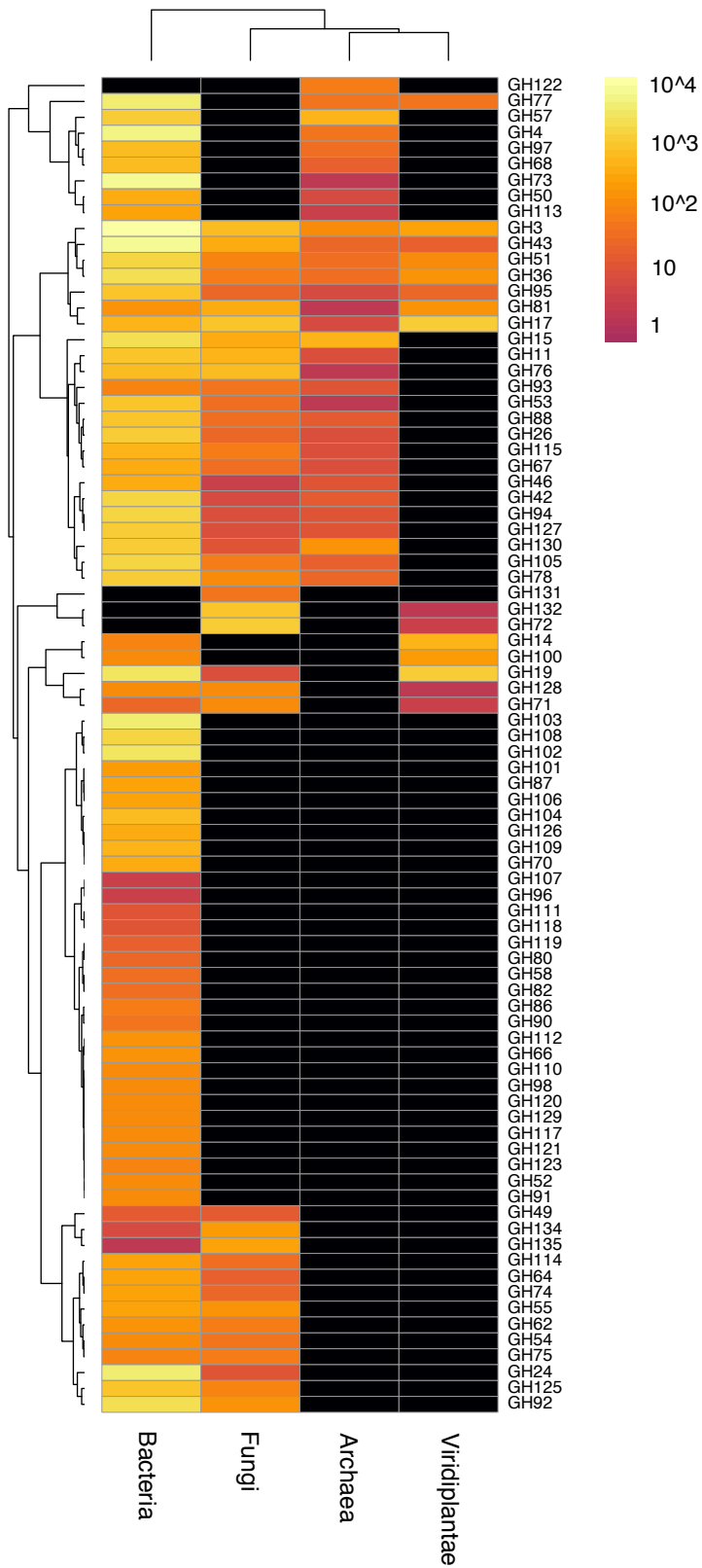

Supplement: Supplementary file 1 — Figure S1. Heatmap depicts 84 GH families identified from CAZy that do not have metazoan representatives. The number of genes within each GH family and taxon are color-coded according to a log10 scale. Dendrograms present clustering of taxa (columns) and GH families (rows) based on hierarchical clustering with Euclidean distance metric and average linkage. Black boxes denote absent members within a particular GH family. (PDF 143 kb) [file 12864_2018_4861_MOESM1_ESM.pdf]

A

CAZy dataset

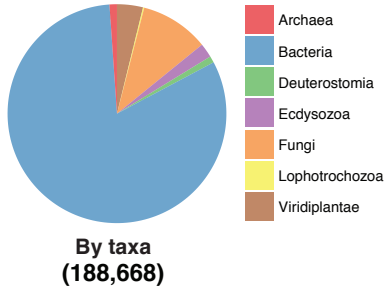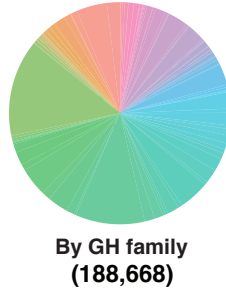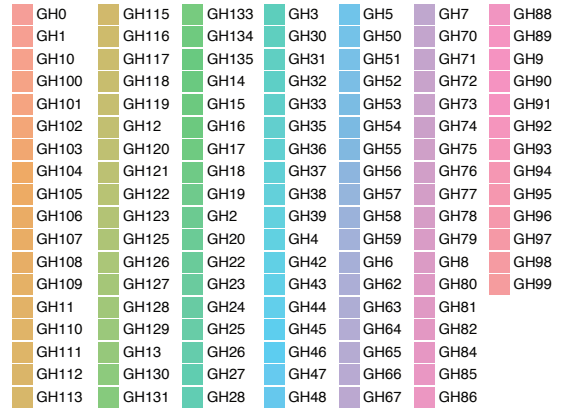

B

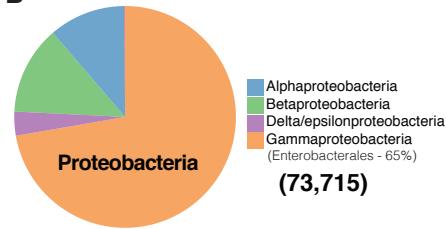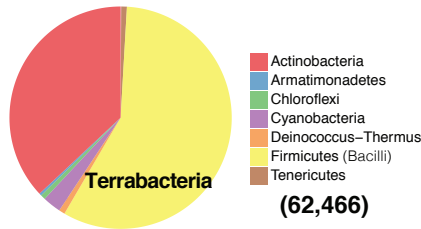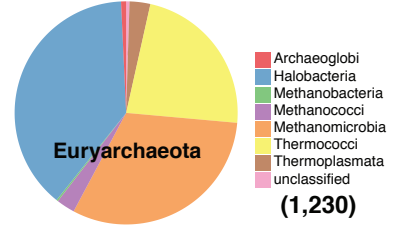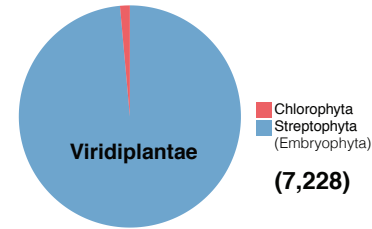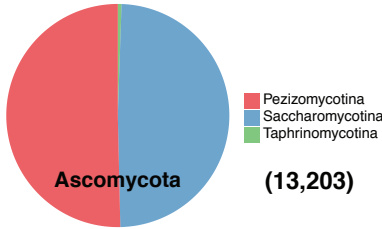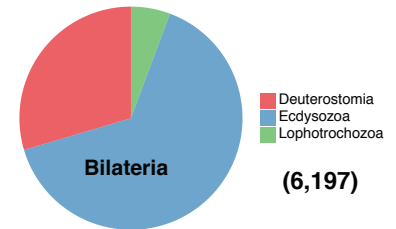

C

Metazoan genomes

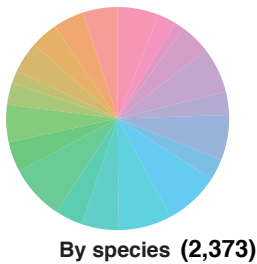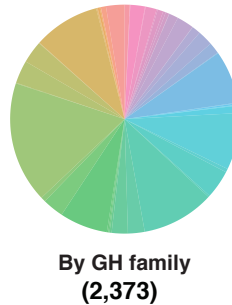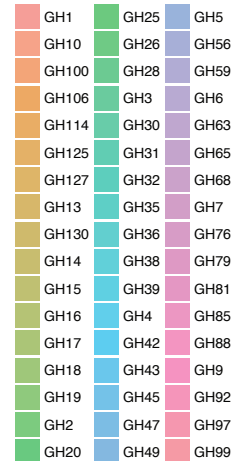

Supplement: Supplementary file 2 — Figure S2. Distribution of GH families retrieved from the CAZy database and identified in this study from metazoan genomes. (A) Pie charts represent the proportion of CAZy GH genes grouped according to taxa and GH families. (B) Proportion of CAZy GH genes within selected taxa are depicted. (C) The proportion of GH families identified from metazoan genomes are represented as pie charts grouped by species and by GH family. Numbers alongside pie charts in parentheses represent the total number of sequences. (PDF 1060 kb) [file 12864_2018_4861_MOESM2_ESM.pdf]

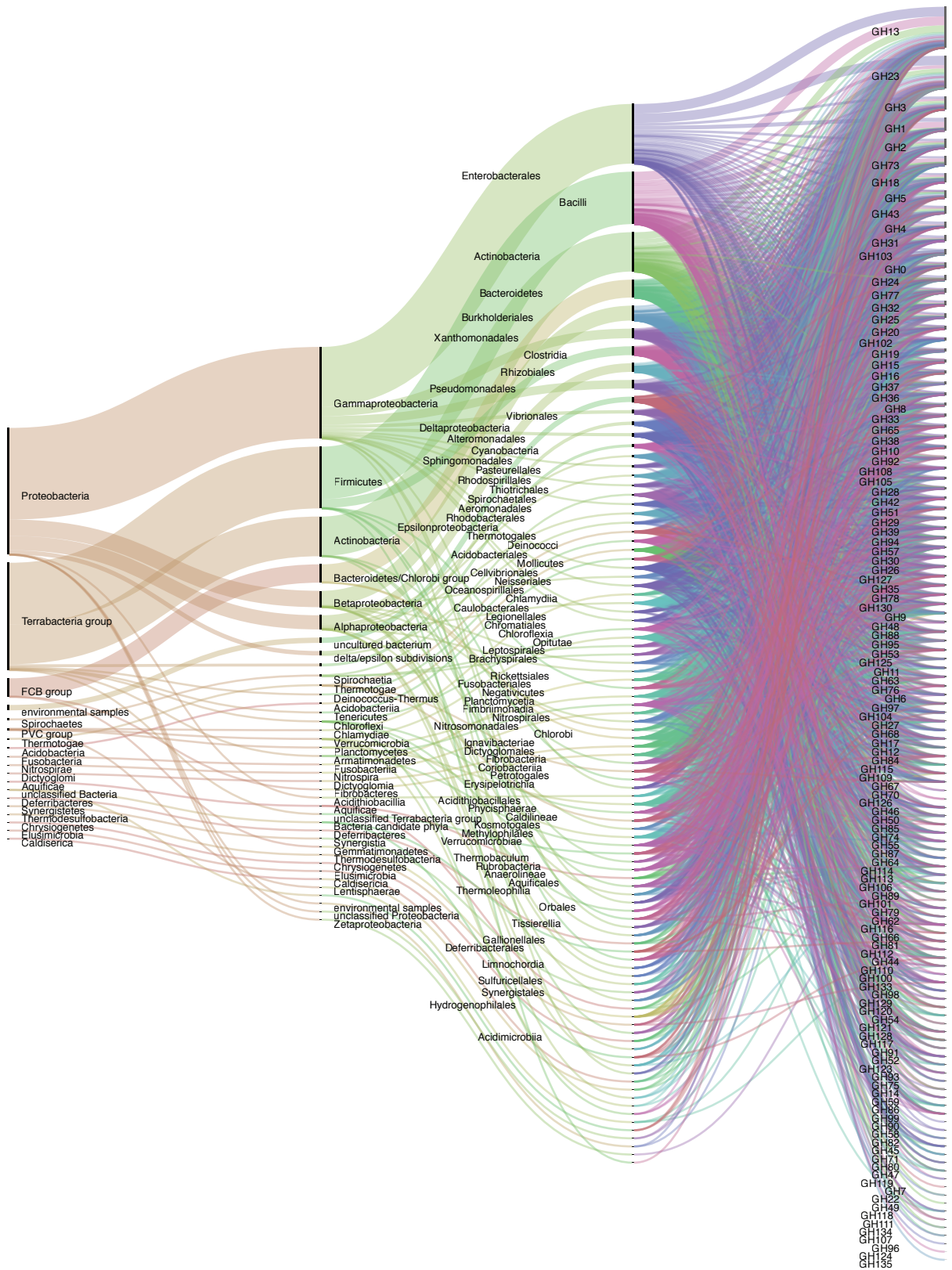

Supplement: Supplementary file 3 — Figure S3. Taxonomic Sankey diagram of CAZy glycoside hydrolases from Bacteria. (PDF 2021 kb) [file 12864_2018_4861_MOESM3_ESM.pdf]

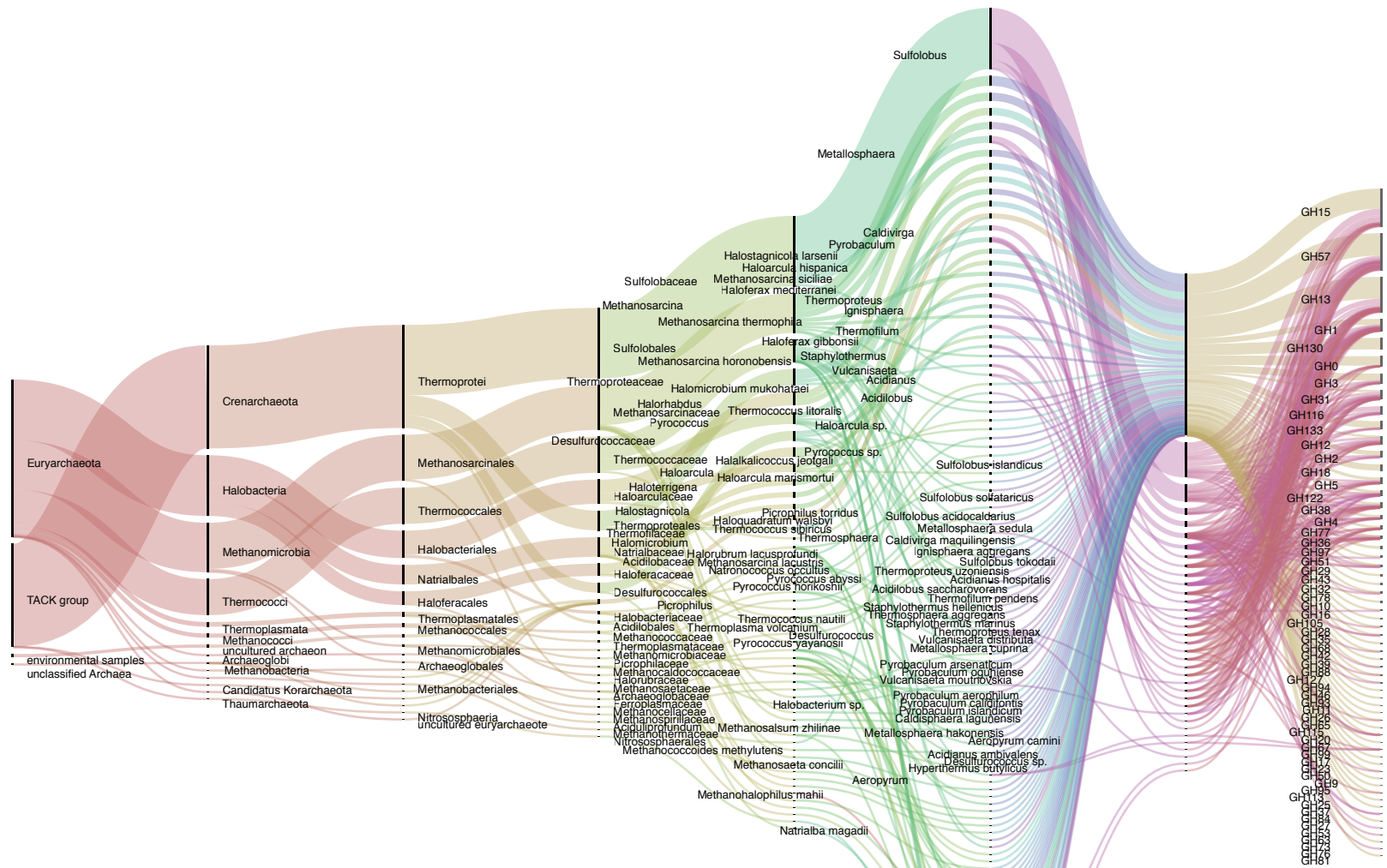

Supplement: Supplementary file 4 — Figure S4. Taxonomic Sankey diagram of CAZy glycoside hydrolases from Archaea. (PDF 869 kb) [file 12864_2018_4861_MOESM4_ESM.pdf]

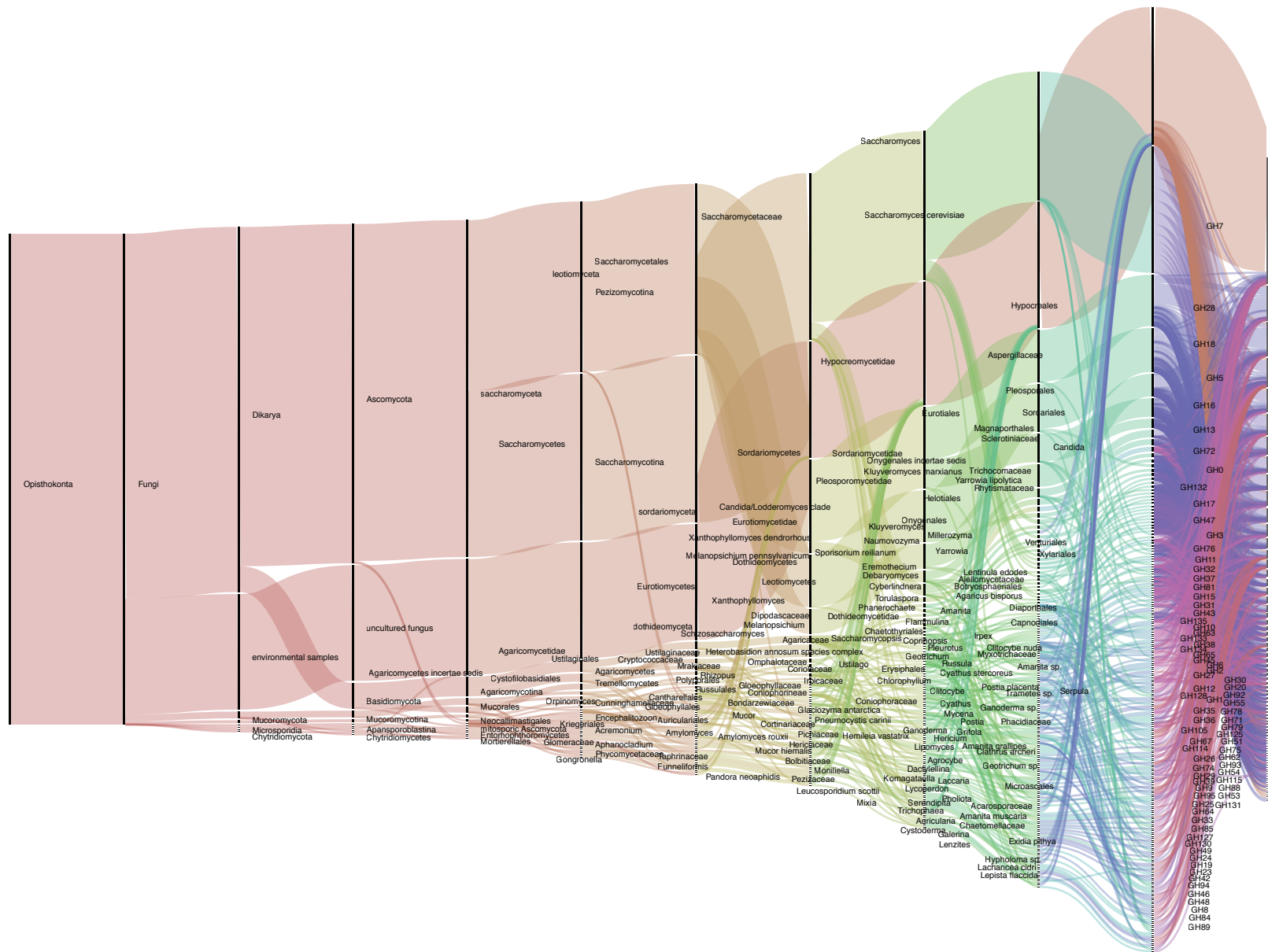

Supplement: Supplementary file 5 — Figure S5. Taxonomic Sankey diagram of CAZy glycoside hydrolases from Fungi. (PDF 1716 kb) [file 12864_2018_4861_MOESM5_ESM.pdf]

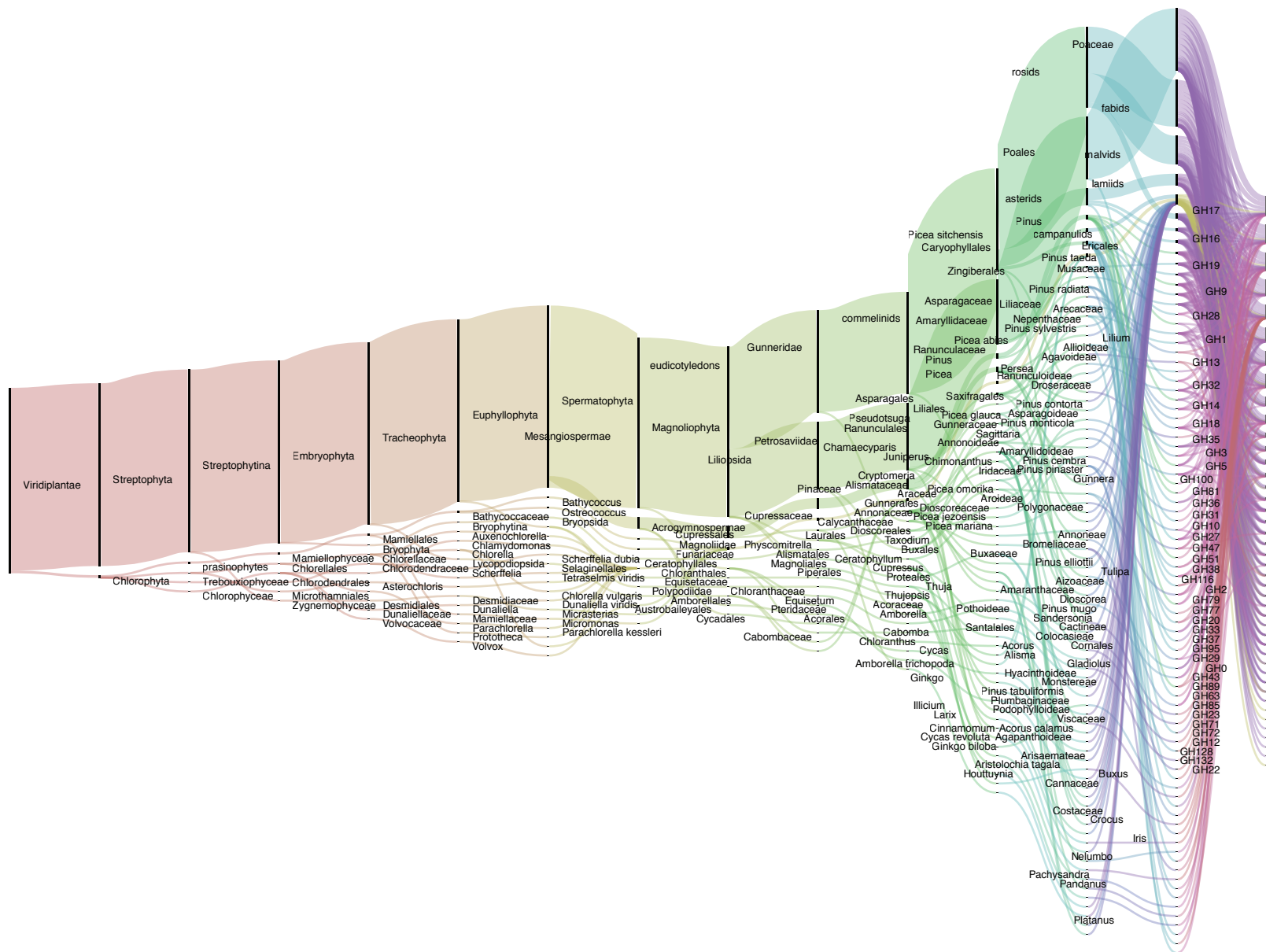

Supplement: Supplementary file 6 — Figure S6. Taxonomic Sankey diagram of CAZy glycoside hydrolases from Viridiplantae. (PDF 997 kb) [file 12864_2018_4861_MOESM6_ESM.pdf]

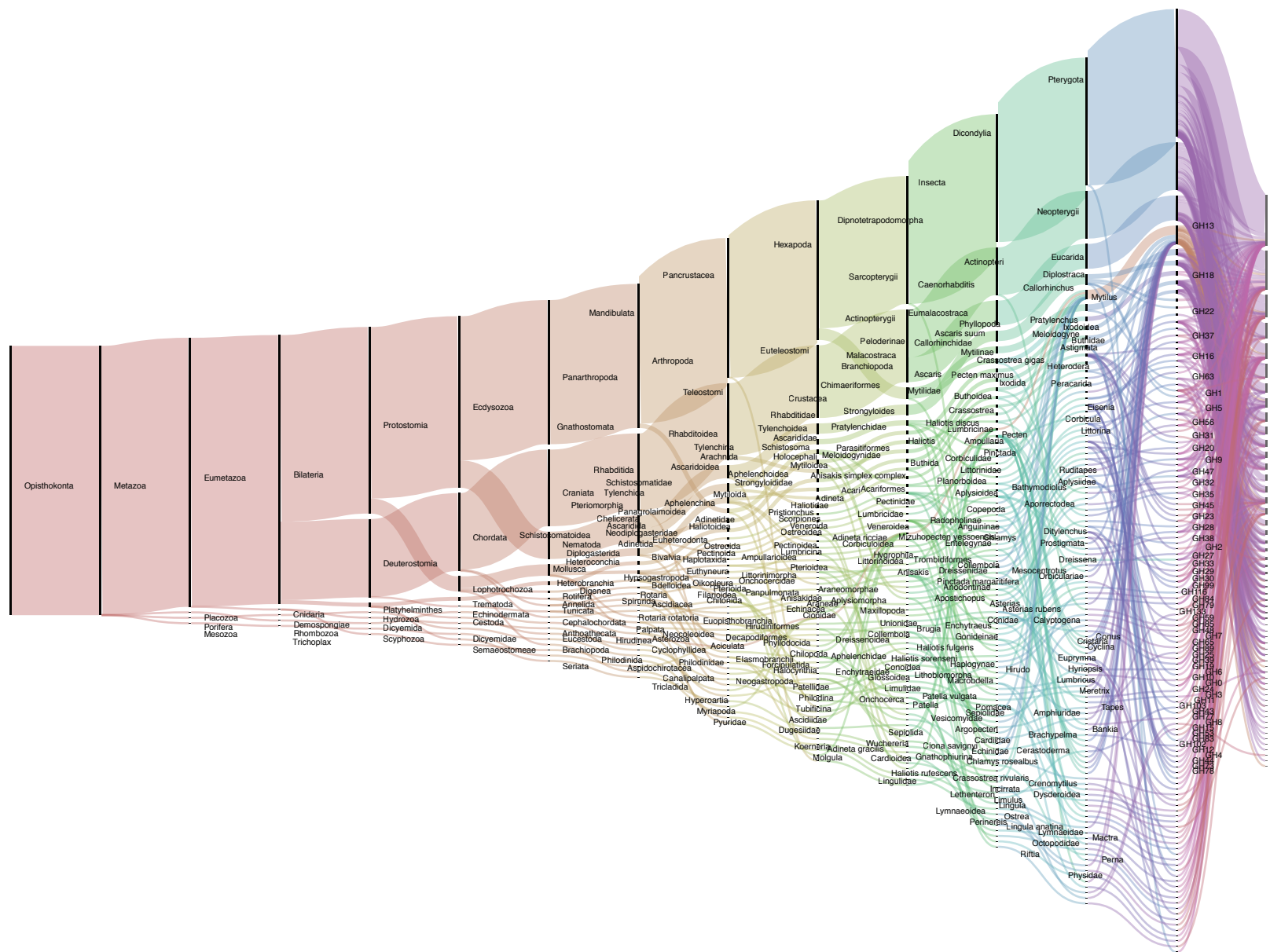

Supplement: Supplementary file 7 — Figure S7. Taxonomic Sankey diagram of CAZy glycoside hydrolases from Metazoa. (PDF 1211 kb) [file 12864_2018_4861_MOESM7_ESM.pdf]

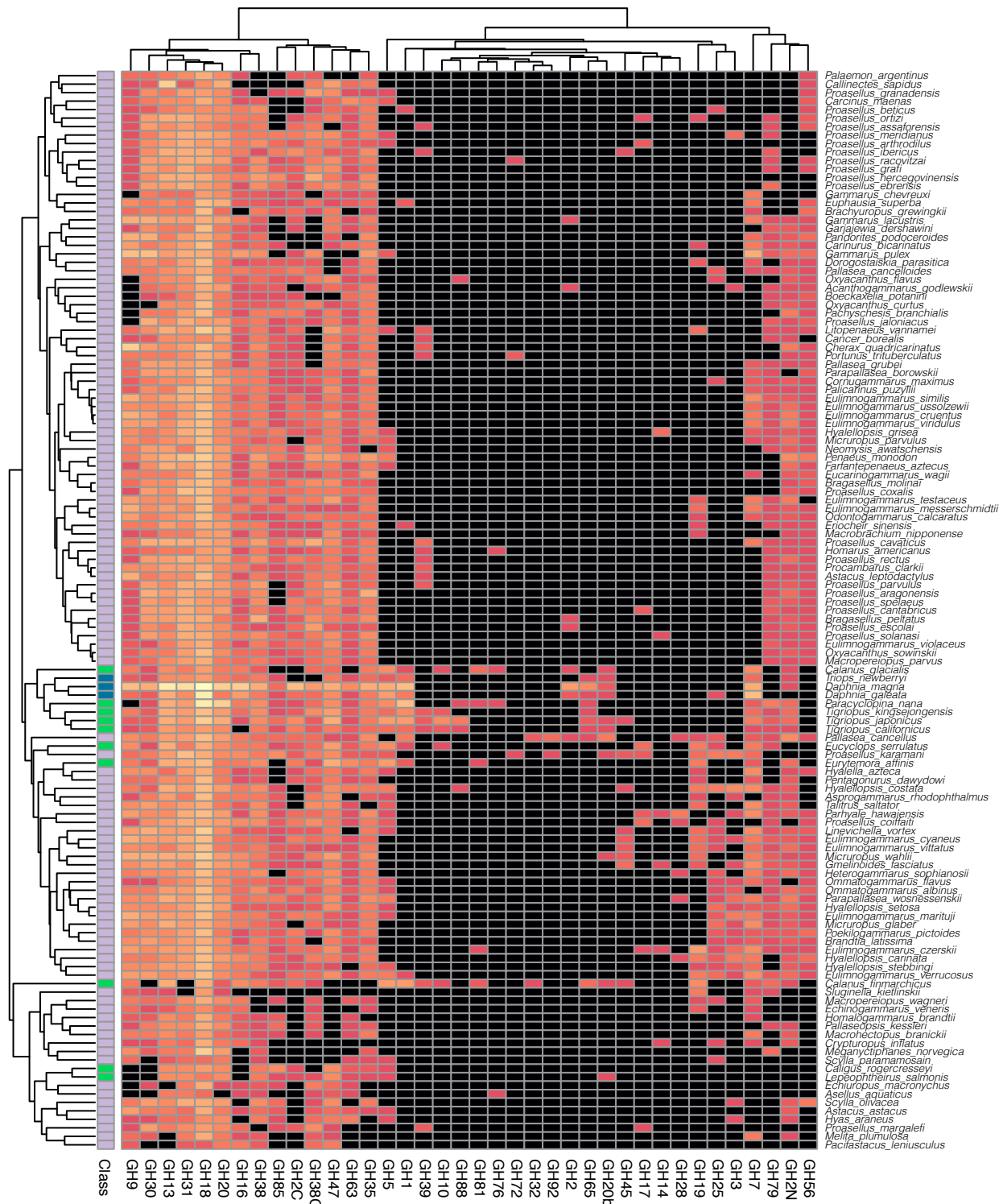

# **Class**

- Branchiopoda
- Copepoda
- Malacostraca

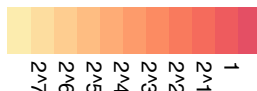

Supplement: Supplementary file 11 — Figure S8. Heatmap illustrating the abundance of GH genes identified from 126 crustacean species. The number of GH genes within each family and taxon are color-coded according to a log2 scale. Dendrograms present clustering of species (rows) and GH families (columns) based on hierarchical clustering with Euclidean distance metric and average linkage. Black boxes denote absent members within a particular GH family. (PDF 441 kb) [file 12864_2018_4861_MOESM11_ESM.pdf]
